# Successful Use of Difelikefalin in Severe Chronic Kidney Disease–Associated Pruritus in a Patient With Complex Etiological Contributors: A Case Report

**DOI:** 10.1155/crin/6626611

**Published:** 2025-02-25

**Authors:** Johannes M. Werzowa, Margit Hemetsberger

**Affiliations:** ^1^First Medical Department, Clinical Division of Nephrology and Dialysis, Hanusch-Krankenhaus, Vienna, Austria; ^2^Department of Medical Writing, Hemetsberger Medical Services, Vienna, Austria

**Keywords:** case report, chronic kidney disease, difelikefalin, hyperparathyroidism, uremic pruritus

## Abstract

**Introduction:** Chronic kidney disease–associated pruritus (CKD-aP) is a frequently experienced, unpleasant skin condition. Difelikefalin, an agonist of the kappa opioid receptor, is indicated for the treatment of moderate-to-severe CKD-aP in adult patients on hemodialysis. Reports of the effectiveness of difelikefalin in complex patient cases encountered in routine clinical practice are rare.

**Case Presentation:** The presented patient had a complex interplay of morbidities, most notably diabetes mellitus type 2, tertiary hyperparathyroidism, end-stage renal disease (ESRD), and CKD-associated mineral bone disease (CKD-MBD), all of which are associated with the development and severity of CKD-aP. The patient's CKD-aP was resistant to H_1_-receptor antagonists and gabapentin and showed no improvement after parathyroidectomy. Treatment with difelikefalin rapidly and sustainably improved symptoms, with a brief recurrence of itching toward the end of each long interdialytic interval. Apart from a short episode of vertigo at the initiation of treatment, no adverse events were observed over the long duration of treatment (currently more than 2.5 years).

**Conclusion:** In a patient with longstanding conditions and multiple comorbidities, difelikefalin showed sustained effectiveness against H_1_-receptor antagonist– and gabapentin–resistant CKD-aP. Difelikefalin was well tolerated over the long term.

## 1. Background

Chronic kidney disease–associated pruritus (CKD-aP) is a very unpleasant skin condition frequently experienced by patients with end-stage renal disease (ESRD). According to the Dialysis Outcomes and Practice Patterns Study (DOPPS), 37% of hemodialysis patients feel moderately (18%), severely (12%), or extremely (7%) affected by pruritus [1], and symptoms have been shown to be chronic in most affected patients [2]. In a recent cohort study from the Austrian Dialysis and Transplant Registry, a CKD-aP prevalence of 14% for at least moderate and 4% for severe pruritus was extrapolated from the observed hemodialysis patients [3]. However, CKD-aP has been observed not only in patients with ESRD but also in approximately one-third of patients with earlier, nondialysis stages of CKD [4].

The location, duration, severity, and perception of CKD-aP vary from person to person. The condition is often bilaterally symmetrical and can be localized or generalized. Itching may periodically reoccur several times a day or persist permanently [5, 6]. Visible, secondary skin lesions may be present, ranging from scratch marks to prurigo nodularis to scarring [7–9]. CKD-aP often occurs along with other physical and psychoemotional symptoms, including poor sleep quality, depression, fatigue, and pain [10, 11]. The DOPPS reported a 1.2-fold higher cause-independent mortality rate and a 1.4-fold higher infection-related mortality rate in patients with severe CKD-aP than in patients who were not affected by pruritus [1].

The pathophysiology of CKD-aP is not yet fully understood. This condition is commonly described as a consequence of the accumulation of uremic toxins in the skin and subsequent peripheral neuropathy, immune system dysregulation, and/or unbalanced activation of mu and kappa opioid receptors [12]. However, these mechanisms appear to be involved to varying degrees in different subsets of patients. In many patients, symptoms of itching improve only transiently or not at all when highly efficient hemodialysis (with increasing Kt/V and control of serum calcium, intact parathyroid hormone (iPTH), or phosphate) is initiated [12]. Evidence suggests that using gabapentin and pregabalin to treat peripheral neuropathy pain may improve CKD-aP [13–15]. However, reports have indicated adverse neurological treatment-related events, such as mental status deterioration or an increased propensity to fall [16, 17]. The administration of antihistamines, although commonly used to treat chronic itching, is ineffective against CKD-aP because the sensation of CKD-aP is transmitted by a pathway of nonhistaminergic itch fibers [12, 18, 19].

Difelikefalin, a kappa opioid receptor agonist, was specifically developed to address the imbalance in the endogenous opioid system, i.e., the overexpression of mu opioid receptors and concomitant downregulation of kappa opioid receptors [20]. Kappa opioid receptor activation on peripheral sensory neurons and immune cells reduces itching and produces immunomodulatory, anti-inflammatory effects [12, 20]. Difelikefalin was first approved in the European Union in April 2022 and is indicated for the treatment of moderate-to-severe CKD-aP in adult patients on hemodialysis [20].

The rapid, sustained efficacy of difelikefalin was demonstrated in the randomized, controlled clinical KALM-1 and KALM-2 trials, in which the results were consistent across diverse populations [21–23]. However, reports of the effectiveness of difelikefalin in complex patient cases encountered in routine clinical practice are still rare. Therefore, we present the case of a complex patient with severe H_1_-receptor antagonist- and gabapentin-refractory CKD-aP, tertiary hyperparathyroidism, and associated bone disease who rapidly and sustainably responded to treatment with difelikefalin.

## 2. Case Presentation

The female patient (year of birth: 1980; height: 160 cm; weight: 57 kg; smoker) initially presented at the clinic in July 2016 following her hospitalization for a knee injury. A routine laboratory workup at the orthopedics department revealed a Modification of Diet in Renal Disease (MDRD)–estimated glomerular filtration rate (eGFR) of 30 mL/min per 1.73 m^2^ and hypercalcemia (total serum calcium 3.6 mmol/L), indicating possible CKD. A sonographic workup revealed suspected kidney concrement.  Table 1 shows comorbidities and risk factors present in the patient.

With an iPTH level of 7200 pg/mL (reference range: 12–88 pg/mL), primary hyperparathyroidism was suspected. Methoxy-isobutyl-isonitrile (MIBI) scintigraphy of the thyroid revealed MIBI retention in the area of the left caudal thyroid pole. A kidney biopsy showed nephrocalcinosis, severe interstitial fibrosis, and tubular atrophy. The adenoma of the right parathyroid gland, which caused the primary hyperparathyroidism, was surgically removed in August 2016. After surgery, the levels of serum calcium and iPTH rapidly improved to approximately 2 mmol/L and 150–600 pg/mL, respectively. The creatinine values fluctuated between 2 and 3 mg/dL over a longer period of time.

In June 2019, diabetes mellitus type 2 was diagnosed, and insulin treatment was initiated shortly thereafter due to high HbA1c values (approx. 8%) despite oral antidiabetic therapy.

**Figure 1:**
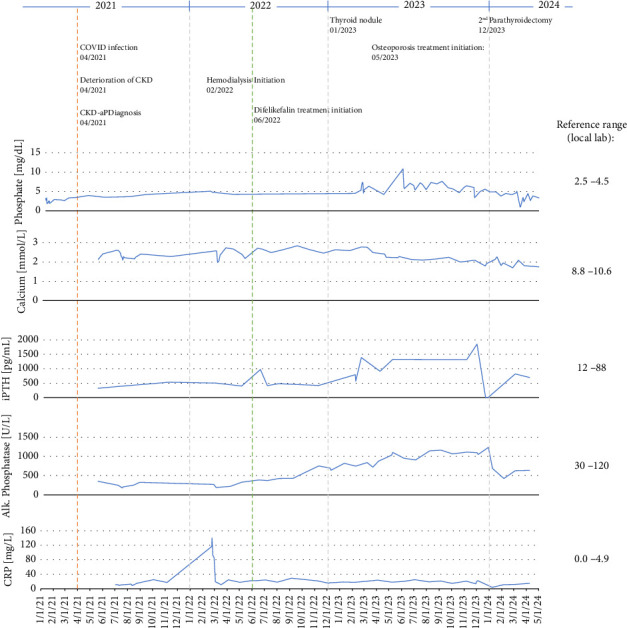
Relevant laboratory parameters and clinical events over time. Alk., alkaline; CKD, chronic kidney disease; CKD-aP, chronic kidney disease–associated pruritus; iPTH, intact parathyroid hormone. The orange line depicts the time of first appearance of CKD-aP symptoms in April 2021. The green line depicts the time at which difelikefalin was initiated in June 2022.

In April 2021, significant deterioration of renal function and electrolytes (hypokalemia and hyponatremia) was observed following severe COVID-19 infection (the patient was not vaccinated). The first observation of CKD-aP occurred during the COVID-19 infection. In August 2021, a Cimino shunt was placed in the left forearm. Chronic hemodialysis was initiated in March 2022. The patient was hyperphosphatemic and therefore initiated a phosphate binder (sevelamer) at the start of hemodialysis. The patient suffered from moderate pruritus at that time.  Figure 1 shows the patient's relevant laboratory parameters and clinical events over time from the start of CKD-aP. The initiation of hemodialysis led to a transient improvement in pruritus intensity, but by May 2022, the condition deteriorated, with a worst-itch numerical rating scale (WI-NRS) of 8-9 and a self-assessed disease severity (SADS) level C. Itching was worst during the night. H_1_-receptor antagonist therapy (diphenhydramine and cetirizine) and gabapentin did not improve symptoms. Topical treatment, including emollients and a polidocanol-containing ointment, also did not provide any relief, and the patient showed massive scratches, especially on the upper extremities.

In June 2022, an early access program in Austria allowed treatment initiation with intravenous difelikefalin at 0.5 mg/kg three times weekly at the end of each dialysis session. The patient's relevant laboratory measurements nearest to initiation of difelikefalin were as follows: iPTH 973 pg/mL, serum calcium 2.60 mmol/L, albumin 3.9 g/dL, HbA1c 6.7%, alkaline phosphatase 386 U/L, and phosphate 2.21 mmol/L.

The patient experienced mild vertigo after the first two to three administrations but then experienced no further side effects. Pruritus decreased significantly within the first two weeks of therapy with WI-NRS 0 and SADS A. Although CKD-aP generally resolved after the initiation of difelikefalin, the patient continued to experience mild pruritus at the end of the long interdialytic interval over the weekends; these symptoms reliably disappeared after the administration of difelikefalin at the end of the first weekly dialysis session each Monday. The patient's dialysis efficacy was adequate (Kt/V > 1.3) at all times. The C-reactive protein (CRP) level was constantly elevated.

Concomitantly, iPTH levels continuously increased to 1200 pg/mL despite treatment with the calcimimetic etelcalcetide (12.5 mg intravenously at the end of each dialysis session). Sonographic examination of the right thyroid revealed a hypoechogenic structure at the dorsocaudal margin of the right thyroid lobe suggestive of a parathyroid adenoma. Scintigraphic examination confirmed the diagnosis of tertiary hyperparathyroidism. A dual-energy X-ray absorptiometry (DXA) scan conducted in April 2023 revealed severe osteoporosis with T scores of −1.7 in the lumbar spine, −3.0 in the femoral neck, and −5.1 in the radius, which is typical of bone disease associated with very high iPTH levels [24]. Bone-specific alkaline phosphatase levels were elevated to 719 U/L (reference range: 35–105 U/L), with an increased fraction of the bone-specific isoform (80%, reference range: 20%–74%). Antiresorptive therapy with alendronate (35 mg orally once weekly) was initiated in May 2023. Surgical resection of the parathyroid adenoma with autotransplantation of gland tissue to the right forearm was conducted in December 2023. After the operation, the iPTH level decreased sharply to 4 pg/mL, and the patient developed severe hypocalcemia. She therefore received calcitriol and oral calcium supplements. Alendronate was discontinued. Phosphate binder use was stopped upon normalization of the phosphate levels. The patient reported no improvement in the frequency or intensity of itching at the end of the long dialysis interval after parathyroidectomy. Currently (January 2025), she is still receiving difelikefalin at the initial dose to control her pruritus without experiencing adverse effects. The patient still has residual kidney function with no need for ultrafiltration to maintain euvolemia.

## 3. Discussion and Conclusions

This is the case of a patient with a complex interplay of morbidities, most notably diabetes mellitus type 2, tertiary hyperparathyroidism, ESRD, and CKD-associated mineral bone disease (CKD-MBD). There is evidence that all of these morbidities are associated with the development and severity of CKD-aP [5, 25–28]. In a cohort study of 1951 nondialysis CKD patients, diabetes, elevated serum iPTH, and reduced eGFR, among other factors, were associated with a greater risk of developing CKD-aP. An incremental reduction of eGFR of 10 mL/min per 1.73 m^2^ was associated with a hazard ratio (HR) for CKD-aP of 1.16 (95% confidence interval (CI): 1.10–1.23). Serum phosphate was not associated with incident CKD-aP, whereas low serum calcium (< 9 mg/dL) was correlated with a reduced incidence of CKD-aP [4]. A recent cross-sectional study of 6221 patients also revealed that diabetes, as well as elevated phosphate and iPTH levels, significantly correlated with increased CKD-aP severity [29]. An Austrian Dialysis and Transplant Registry analysis revealed that a CRP or iPTH level above the sample median (CRP > 0.5 mg/dL; iPTH > 300.5 pg/mL) was associated with more severe pruritus (CRP: odds ratio (OR) 1.61, 95% CI: 1.07–2.43; PTH: OR 1.50, 95% CI: 1.00–2.27). Absolute serum calcium above the cohort mean (> 8.63 mg/dL) was associated with a lower risk for moderate-to-severe pruritus (OR 0.62, 95% CI: 0.41–0.93), but statistical significance was lost when calcium values were corrected for serum albumin. Serum phosphate above the cohort mean (> 5.53 mg/dL) was not significantly associated with an increased risk of moderate-to-severe pruritus (OR 1.19, 95% CI: 0.79–1.78, *p*=0.433) [3]. An association between elevated iPTH levels and CKD-aP severity was also reported in other studies [5, 26–28]. The disappearance of uremic pruritus after parathyroidectomy was described in the 1960s [30, 31] and was later confirmed in a study by Chou et al. [32] in patients with pruritus and secondary hyperparathyroidism.

However, there is also evidence indicating a lack of correlation between CKD-MBD and pruritus. The DOPPS study did not find any links between the severity of CKD-aP and the levels of iPTH, calcium, or phosphate [33]. In the phase 3 KALM-1 and KALM-2 trials of difelikefalin, serum phosphate levels did not correlate with CKD-aP severity or with response to difelikefalin [21, 23]. Local injection of PTH into the skin of dialysis patients also did not induce pruritus at the injection site [34].

The presented patient had persistently high levels of iPTH, alkaline phosphatase, phosphate, CRP, and creatinine, all of which further deteriorated when kidney function worsened in the spring and early summer of 2021 (Figure 1). Around this time, the patient first experienced pruritus. When hemodialysis started in February 2022, pruritus worsened, and the patient quickly required specific treatment for CKD-aP upon the appearance of severe scratch marks. The patient's pruritus did, however, not improve substantially when H_1_-receptor antagonists and subsequently gabapentin were used. Therefore, treatment with difelikefalin was initiated, and symptoms quickly and sustainably improved. However, after each long interdialytic interval, a recurrence of the itching was observed, owing to the mean half-life of difelikefalin of 38 h in hemodialysis patients [35]. Currently, the duration of difelikefalin treatment is more than 2.5 years, with no notable adverse effects apart from a short and mild episode of vertigo at the time of initiation. Parathyroidectomy, followed by a substantial decrease in iPTH levels, did not alleviate pruritus.

It was hypothesized that overstimulation of central mu opioid receptors, antagonism of peripheral kappa opioid receptors, or an imbalance of stimulation and antagonism of mu and kappa opioid receptors causes itching [20, 36, 37]. The mechanism of action of difelikefalin via the kappa opioid receptor [12, 20] is thus directed at a mechanism of itch sensation that is independent of the comorbidities present in the patient, notably diabetes mellitus type 2, tertiary hyperparathyroidism, ESRD, and CKD-MBD. This may explain the sustained effectiveness of difelikefalin throughout the patient's complex clinical history.

In conclusion, this patient case shows sustained effectiveness and tolerability of difelikefalin in a complex, H_1_-receptor antagonist and gabapentin–resistant patient with longstanding conditions and multiple relevant comorbidities. Within two weeks of difelikefalin treatment, the WI-NRS score improved from 8-9 to 0 and the SADS level improved from C to A. Difelikefalin acts via a pathway independent of CKD-associated causes of pruritus, such as elevated iPTH, calcium, CRP, and possibly phosphate, and thus seems to be able to sustain its effectiveness despite recurring deterioration of these conditions.

## Figures and Tables

**Table 1:** Comorbidities and risk factors.

Comorbidity	Comment
Nephrolithiasis	• First diagnosed in June 2015

Nephrocalcinosis, severe interstitial fibrosis, tubular atrophy	• Kidney biopsy in August 2016

Primary hyperparathyroidism	• Parathyroid adenoma
	• Parathyroidectomy left caudal on 1 August 2016

Secondary hyperparathyroidism	• Secondary hyperparathyroidism in connection with chronic renal insufficiency
	• Large single-gland disease on the right dorsal
	• Second parathyroidectomy on 6 December 2023 with autotransplantation

Diabetes mellitus type 2	• First diagnosed in June 2019
	• Insulin dependent

COVID-19 infection	• April 2021, resolved

End-stage renal disease	• Chronic hemodialysis initiated March 2022

Uremic pruritus	• First experienced in April 2021

Severe osteoporosis	• DXA scan in April 2023
	• Alendronate 35 mg/week p.o. from May 2023 to December 2023

Chronic hyponatremia and hypokalemia	• Potential Bartter syndrome or Gitelman syndrome was excluded through genetic testing

Nicotine abuse	

## Data Availability

Physician–patient privilege protects the data not included in this article, and the patient's consent is required to make them available. Further inquiries can be directed to the corresponding author.
